# Enzyme-linked immunoassay for dengue virus IgM and IgG antibodies in serum and filter paper blood

**DOI:** 10.1186/1471-2334-6-13

**Published:** 2006-01-25

**Authors:** Thanh Nga T Tran, Peter J de Vries, Lan Phuong Hoang, Giao T Phan, Hung Q Le, Binh Q Tran, Chi Mai T Vo, Nam V Nguyen, Piet A Kager, Nico Nagelkerke, Jan Groen

**Affiliations:** 1 Division of Infectious Diseases, Tropical Medicine and AIDS, Academic Medical Center, P.O. Box 22700, 1100 DE Amsterdam, the Netherlands Amsterdam, the Netherlands; 2 Department of Microbiology, Cho Ray Hospital, 102 B Nguyen Chi Thanh, Ho Chi Minh City, Vietnam; 3 Department of Tropical Diseases, Cho Ray Hospital, 102 B Nguyen Chi Thanh, Ho Chi Minh City, Vietnam; 4 Binh Thuan Malaria and Goiter Control Center, 133A Hai Thuong Lan Ong, Phan Thiet, Vietnam; 5 Dept of Community Medicine, United Arab Emirates University, P.O. Box 17666 Al Ain, United Arab Emirates; 6 Department of Clinical Virology, Erasmus Medical Center Rotterdam, The Netherlands; 7 Department of Microbiology and Virology, Focus Diagnostics, Cypress CA, USA

## Abstract

**Background:**

The reproducibilty of dengue IgM and IgG ELISA was studied in serum and filter paper blood spots from Vietnamese febrile patients.

**Methods:**

781 pairs of acute (t0) and convalescent sera, obtained after three weeks (t3) and 161 corresponding pairs of filter paper blood spots were tested with ELISA for dengue IgG and IgM. 74 serum pairs were tested again in another laboratory with similar methods, after a mean of 252 days.

**Results:**

Cases were classified as no dengue (10 %), past dengue (55%) acute primary (7%) or secondary (28%) dengue. Significant differences between the two laboratories' results were found leading to different diagnostic classification (kappa 0.46, p < 0.001). Filter paper results correlated poorly to serum values, being more variable and lower with a mean (95% CI) difference of 0.82 (0.36 to 1.28) for IgMt3, 0.94 (0.51 to 1.37) for IgGt0 and 0.26 (-0.20 to 0.71) for IgGt3. This also led to differences in diagnostic classification (kappa value 0.44, p < 0.001) The duration of storage of frozen serum and dried filter papers, sealed in nylon bags in an air-conditioned room, had no significant effect on the ELISA results.

**Conclusion:**

Dengue virus IgG antibodies in serum and filter papers was not affected by duration of storage, but was subject to inter-laboratory variability. Dengue virus IgM antibodies measured in serum reconstituted from blood spots on filter papers were lower than in serum, in particular in the acute phase of disease. Therefore this method limits its value for diagnostic confirmation of individual patients with dengue virus infections.

However the detection of dengue virus IgG antibodies eluted from filter paper can be used for sero-prevalence cross sectional studies.

## Background

Dengue fever is mostly a rather undifferentiated febrile disease with non-specific signs and symptoms, and molecular and serological tests are can be used to confirm the clinical diagnosis.

Serological confirmation of dengue has become available to many laboratories by commercially available assays. Dengue serology is applied in different settings, such as for surveillance, in health care facilities in endemic areas and in travel clinics in non-endemic areas[1] The applicability and quality of serological tests in dengue endemic regions has to be judged against a background of potential cross reactivity with other flavi-viruses, difficulties in distinguishing primary from secondary infections and technological problems related to the fact that most dengue endemic regions are relatively poor of resources.

Enzyme linked immuno-assay (ELISA) is a convenient technique which allows laboratories to test numerous samples in a short time. Different assays are available on commercial basis. Dengue IgM capture ELISA (MAC-ELISA) and IgG ELISA are both sensitive and specific assays for detection of dengue antibodies but distinction from other endemic flavivirusses is important, in Southeast Asia especially Japanese encephalitis B (JEB) virus[2,3]

Assays that apply antigen from dengue virus type (DEN) 1 through 4, have a high sensitivity and specificity, typically ranging from approximately 90 to 100 %, but do not discriminate between the four serotypes[3,4] The distinction between primary and secondary infections is now mainly based on recognizing the different IgM and IgG responses to primary and secondary infections in two samples taken from a febrile patient in the acute stage of disease and after convalescence[5,6]

Often however, the sample cannot be tested at the spot and needs to be stored and transported before analysis. Blood spots on filter paper are often used as an alternative to collecting serum samples. Dried in the air, they can be stored easily. Filter papers are used for several purposes, such as screening of newborns for congenital hypothyroidism and phenylketonuria (PKU), DNA diagnostics and detection of antibodies[7,8] They are also used for ELISA detection of antibodies against dengue and other viral infections. However, although several technical aspects have been studied, including the duration and temperature of storage, experience is limited[6,9-13]

In this study we investigated several aspects of the variability in dengue IgM and IgG ELISA results in serum and blood spots on filter paper, and their decay during storage, from febrile patients who presented at primary health care facilities in southern Vietnam, an area with a high incidence of dengue virus infections which is also endemic to JEB virus.

## Methods

The study was performed in twelve, not adjacent, commune health posts and the out patient clinic of the provincial malaria station of Binh Thuan, a province in southern Vietnam. Patients presenting with acute undifferentiated fever (AUF) at these primary health care facilities were included in this study. AUF was defined as any febrile illness of duration less than 14 days, confirmed by an axillary temperature ≥ 38.0°C, without any indication for either severe systemic or organ specific disease. Malaria was excluded by microscopic examination of a thick blood smear.

Record forms were filled out for all AUF patients, recording patient identifiers, duration of disease, signs and symptoms. Data and blood were collected on presentation (t0) and all included subjects were asked to come back after 3 weeks (t3) for re-assessment and collection of a second blood sample.

### Collection and storage of blood samples

Blood was collected by venapuncture. A few circles of 15 mm, printed on specimen collection paper (cotton linters paper, S&S 903, Schleicher & Shuell, Dassel, Germany), was filled with full blood and left to dry at the air, avoiding exposure to sunlight. They were packed separately in zip-closure nylon bags, with a few silica grains to prevent moisturizing. The remaining blood in the collection tube was left to clot and thereafter centrifuged at 1000 rpm for 15 minutes at the spot. The serum was decanted into two polypropylene vials and stored in a freezer at minus 20°C at the health facilities. The samples were collected once a month by the research team of Cho Ray Hospital and transported to Ho Chi Minh City in a cooling box, filled with ice packs (duration of this travel is approximately 4 hours). In Ho Chi Minh City the serum samples were stored at minus 70°C until analysis. The filter papers were kept in their nylon bags in an air-conditioned room.

### Reconstitution of sample from filter papers

Samples from dried blood filter paper were reconstituted as described previously[14] In brief, two 6 mm disks were punched from the filter paper and soaked in a vial over night at 4°C on an automatic shaker with 0.2 ml of phosphate-buffered saline supplemented with 2% fetal bovine serum. The following day the supernatant was stirred, collected and analyzed, together with the corresponding serum pairs. The resulting sample was treated as a 1:20 dilution of human serum, assuming that the two circles contain approximately 25 μL of blood, corresponding to 10 μl of serum.

### ELISA

IgM and IgG of both t0 and t3 specimens were always tested in the same batch, on the same day. All samples were tested in the laboratory of microbiology of Cho Ray Hospital, Ho Chi Minh City (CRH), Vietnam. A subset of sera was also tested in the Laboratory of Virology, Erasmus Medical Center, Rotterdam, the Netherlands (EMC), by the same persons, following the same protocol and using similar test kits with the same batch numbers. The concentrations of dengue-virus specific IgM antibodies were measured using a commercially available IgM capture ELISA (Dengue Fever Virus IgM Capture ELISA, Focus diagnostics, Cypress, CA, USA), as described previously[3,4] For IgG antibodies an indirect ELISA was used (Dengue Fever Virus IgG Focus diagnostics, Cypress, CA, USA). Both the IgM and IgG ELISAs detect antibodies against an antigen mixture of equal portions of DEN 1–4. The ELISA plates were read by optical density (OD) ELISA plate readers. OD values were measured at 450 nm with 620 nm as a reference. In CRH a Benchmark microplate reader was used (Bio-Rad Laboratories, Inc., Hercules, CA, USA), in the EMC an Anthos Reader 2001 Type 10 500 (Anthos Labtec Instruments, Salzburg, Austria). Serum and the reconstituted filter paper samples, both IgG and IgM, were tested at a final dilution of 1:100 (implying an extra 1: 5 dilution of the filter paper supernatant). Following the instructions of the manufacturer, the OD results were corrected by subtracting the OD values of blank samples which are included in every test kit. The ratio with a standard positive sample, the cut-off calibrator, is expressed as an index value (IV). All samples were tested in duplicate and the mean IVs were used for further analysis. Quality control is provided by testing three control sera, enclosed in every test kit. IV < 1 is considered negative, IV > 1 positive. The value 1 is not specified. In this study we chose to interpret an IV of exactly 1 as positive.

In order to analyze misclassification and agreement, the results were categorized into four diagnoses. A molar increase of dengue antibody concentrations, measured by ELISA, is generally regarded significant[6] In this study a four fold increase of IV was taken as a significant, based on a previous study by Cobelens[15] The IgM concentration on t3, relative to the IgG concentration on t3 was also used as a criterion, recognizing that this ratio between the results of a capture IgM ELISA and direct IgG ELISA is slightly different from the method published by the WHO[6]. Acute primary dengue virus infection was defined as a positive IgM on t3 with an IgM/IgG ratio on t3 greater than one. A positive IgM on t3 with an IgM/IgG ratio on t3 less then one, or a negative IgM reaction on t3 but with a positive IgGt3 and a fourfold molar increase of IgG between t0 to t3 were classified as acute secondary dengue. A negative IgM reaction on t3, a positive IgG on t3 but without a fourfold increase between t0 and t3 was classified as "not acute dengue but past infection", and a case of both negative IgM and IgG on t3 was classified as "no dengue".

### Data analysis

Intra-individual variation of IgG IVs was investigated in the t0 and t3 sera of subjects classified as cases of past dengue or no dengue. ELISA results of serum and filter paper samples were compared by linear regression and calculation of the coefficient correlation. Agreement between the tests done in the two different laboratories and between serum and filter paper results were analyzed by comparing the positive and negative results and the diagnostic classification based on paired serum samples. The decay during storage of serum was analyzed with a linear mixed effects model of IV as dependent variable and the time between sampling and testing blood, t-store, as independent variable, applied to the sera which were tested twice. The decay of filter papers was estimated by ordinary least squares linear regression of the difference between the serum and corresponding filter paper IVs, ΔIV, against t-store. Statistical analysis was done using SPSS (version 11.5, SPSS Inc. Ill.). We choose to present scatter plots, instead of Bland Altman plots, because that also illustrates the distinction between positive and negative results.

The study was approved by the Review Board of the Cho Ray Hospital, Ho Chi Minh City, Vietnam. The study was explained and discussed in meetings with provincial authorities and health post staffs. All patients, (or their parents or guardian) gave their written informed consent (for children from parent or guardian).

## Results

### ELISA results

Complete sets of paired t0 and t3 sera were available of 781 patients with undifferentiated fever. A subset of 73 serum pairs was tested twice in the two different laboratories (IgGt0 was tested twice in 74 sera). Corresponding filter papers of 161 cases were also tested. The number of specimens tested, t-store, and the proportion of positive test results (index value ≥ 1) are shown in table 1. Based on the first test results, 428 (55%) of patients were classified as "past dengue", 54 (7%) as "acute primary dengue", 218 (28%) as "acute secondary dengue" and 80 (10%) as "no dengue".

**Table 1:** Dengue antibody detection by ELISA in serum and reconstituted filter paper blood of Vietnamese febrile patients, repeated tests and storage time of specimens.

	Dengue tests in febrile patients			
	Upon presentation (t0)		After convalescence (t3)	
	
	IgM	IgG	IgM	IgG
Total cases tested	781			
Storage time (days, median, range)	252 (57 – 751) days		228 (36 – 730)	
No. positive sera (index value ≥1)	222 (28%)	673 (86%)	261 (33%)	679 (87%)

No. cases tested twice	73	74	73	73
Median storage time first test (days, range)	114 (57 – 283)		92.5 (36 – 253)	
Median storage time second test (days, range)	280 (225 – 798)		259 (204 – 777)	
No. positive first test	9 (12%)	59 (80%)	17 (23%)	56 (77%)
				
No. positive second tests	23 (32%)	63 (85%)	23 (32%)	61 (84%)
P value (Chi Square test)	<0.001	<0.001	<0.001	<0.001

No. of combined sera and blood spots on filter paper tested	161			
Median storage time (days, range)	283 (139 – 795)		262 (118 – 774)	
No. positive sera	46 (29%)	121 (75%)	89 (55%)	128 (80%)
No. positive filter papers	8 (5%)	97 (60%)	55 (34%)	123 (76%)
P value (Chi Square test)	<0.0001	< 0.0001	<0.0001	<0.0001
Kappa value	0.191	0.391	0.567	0.549

t3: three weeks after t0.

Intra-individual variation of IgG, expressed as the correlation between serum IgGt0 and IgGt3 IVs, in cases classified as "past dengue" and "no dengue" was high (R^2 ^= 0.861). In four (0.7%) cases of "past dengue" IgGt0 was negative (index value < 1) whereas IgGt3 was positive. Of the cases classified as "no dengue" (IgG3<1), 19 (24%) had a positive IgGt0.

### Inter-laboratory variation

The inter-laboratory variation was higher. Results of the repeated measurements in the two different laboratories were significantly different for IgGt0 (mean IV at first test: 3.0; at second test: 5.1; mean (95% CI) difference: 2.1 (1.5 to 2.6); p value < 0.001) as well as for IgGt3 (mean IV at first test: 3.3; at second test: 5.8; mean (95% CI) difference: 2.5 (1.8 to 3.2); p value < 0.001). At higher values of the index value, the variability increased. Although this did not lead to significant misclassification into positive or negative it did affect the diagnostic classification based on paired samples. Twelve cases classified as "no dengue" or "past dengue" in the one laboratory were classified as acute dengue in the other and vice versa in 6 cases (Overall Chi square 52, kappa value 0.46, p < 0.001).

### Comparison of serum and filter papers

Comparison between filter paper and serum showed that IVs tended to be lower and more variable in reconstituted filter paper than in the corresponding serum (Figure 2). The mean (95% CI) difference with the IV of the corresponding serum was 0.99 (0.67 to 1.31) for IgMt0, 0.82 (0.36 to 1.28) for IgMt3, 0.94 (0.51 to 1.37) for IgGt0 and 0.26 (-0.20 to 0.71) for IgGt3.

Table 1 shows that on t0 the detection of dengue virus IgM from filter paper eluate is not very sensitive, compared to serum. The agreement between filter paper eluates and serum was substantially higher. Of the 89 cases with a positive IgMt3 in serum, the corresponding filter paper eluates were positive in 54 (61%) cases. For dengue virus IgG the agreement was higher on t0 and also increased on t3. Of the 128 sera positive on t3, 113 (88%) corresponding filter paper eluates were positive.

The consequences for the diagnostic classification of cases are shown in Table 2. Differences in classification were in two directions but with filter papers patients were less frequently classified as acute dengue (Chi square 119, kappa value 0.43, p < 0.001).

**Table 2:** Serological diagnosis of dengue, based on ELISA on serum versus ELISA on reconstituted filter paper blood.

Diagnosis based on ELISA on filter paper	Diagnosis based on ELISA on serum				Total
	acute primary	acute secondary	no dengue	past dengue	
Acute primary	12	1	0	0	13
Acute secondary	10	33	0	3	52
no dengue	5	2	17	13	37
past dengue	9	20	4	32	59

Total	36	56	21	48	161^a)^

a) Overall kappa value: 0.43, P value < 0.001

The mean serum/filter paper IV ratios were 4.84 for IgMt0, 6.93 for IgMt3, 2.61 for IgGt0 and 1.70 for IgGt3.The distribution of the IVs of all filter papers was non-Normal and there was a poor linear relationship with their corresponding serum values. Consequently, no simple conversion factor could be determined and correction of the filter paper IVs by multiplying with the mean serum/filter paper IV-ratios did not improve the agreement between the serum and filter paper diagnostic classifications (overall kappa 0.294).

### Effects of storage

The decay of serum Ig during storage was analyzed with mixed effects models, applied to the serum tests which were done two times. Four linear mixed effects models were constructed, with the repeated IgMt0, IgMt3, IgGt0 and IgGt3 index values as the dependent variables and t-store as fixed independent effect. The test identifier, indicating that the two tests were done on different occasions, was entered as cofactor. Also the natural logarithms of the index values were examined. No significant independent effect of the storage time on the index value was observed (mean increase of IV / day (95% CI) for IgM0: -0.0002 (-0.0009 to 0.0005, p = 0.62); IgM3: -0.0004 (-0.0014 to 0.0005, p = 0.39); IgG0: 0.0010 (-0.0009 to 0.0030, p = 0.28); IgG3: 0.0016 (-0.0007 to 0.0039, p = 0.17).

Similar to serum, there was no significant effect of the duration of storing filter papers (Change of ΔIV per day (95% CI) for IgM0: -0.001 (-0.003 to 0.001, p = 0.315); IgM3: -0.0005 (-0.004 to 0.003, p = 0.8); IgG0: 0.001 (-0.002 to 0.004, p = 0.4); IgG3: 0.003 (0.000–0.006, p = 0.052).

## Discussion

The dengue ELISA in this study was performed by the same skilled persons, working in two laboratories, one of which is a virological reference laboratory in an industrialized country (EMC) and another in a resource poor setting (CR), using the same operating procedures and commercially available test assays. The conditions in this study are a realistic reflection of routine diagnostics with commercially available assays in endemic settings.

The diagnostic classification applied in this study is based on the different time course of antibody concentrations after primary and secondary dengue virus infections[6] In the acute stage of secondary infections, IgM may be detected in all patients, but after convalescence, IgM concentrations may be lower or even undetectable[16,17] In this study the second sample was taken more than three weeks after onset of fever. An increase of IgG without detectable IgM is thus compatible with secondary dengue but it may also indicate another flavivirus infection.

Cross reactivity with other flavivirusses is a potential source of error. Test kits are usually validated against a set of well defined patients and healthy individuals. In endemic regions such as Vietnam however, repeated dengue and other flavi-virus infections, such as JEB, are common[1]. Other studies have attempted to enhance the distinction with other flavivirusses by applying higher cut off values, thereby losing sensitivity[2,5] This study did not address cross reactivity with JEB virus but cases with clinically evident encephalitis were excluded. Although the clinical pattern of JEB is different compared to dengue virus infections we cannot completely rule out that some of the reactivity measured in serum and on filter paper was due to the serological cross-reactivity between both viruses

Inter-laboratory disagreement on serological results is not uncommon. The interpretation of serological tests is often difficult and random and systematic errors may occur in the serological confirmation of dengue in endemic as well as in non-endemic areas [18-20] This is partly due to differences between the used test kits but also to other technical aspects, among which the optical density (OD) ELISA plate readers[3] Variability in OD values is usually accounted for by making reference to a control[21] However, the relation between "real" extinction and measured OD values is not always linear, especially not at the extremes of its measurement range. The cut off value, the distinction between positive and negative, is ideally set at a value where most OD ELISA plate readers show linear characteristics. Nonetheless, in this study the inter-laboratory variability also occurred in the range around the cut off index value, 1. Although this was mainly a random error, with almost equal variability within both laboratories, with a small systematic compound (the intercept of the regression lines), it led to differences in classification of positive and negative in the two laboratories. Other potential sources of variability and error, related to laboratory procedures, were not uncovered in this study.

Differences in storage time did not explain the variability in this study, neither for serum nor for filter papers. Other studies have indicated that filter papers should be stored in a refrigerator but that this does not completely prevent a decline of antibody concentrations[9,10] It was also shown that in particular the anti-dengue IgM in filter paper blood spots from secondary cases decreased significantly on storage[10] In this study filter papers were not stored in a refrigerator but in an air-conditioned room. This may be an advantage because it avoids temperature differences and thus condensation of moisture on the papers. In addition, it simulates the reality of using filter papers, usually under conditions where electrical power is not available. We paid special attention to keeping the filter papers dry and found no significant decay of antibody contents.

Filter paper index values tended to be lower than the corresponding serum values. Several studies have shown that the antibody titers measured in serum eluted from filter paper blood were 1 or 2 dilution factors lower compared to serum, especially for IgM antibodies[10,12,22].The ratio of serum and filter paper concentrations is sometimes used as conversion factor but in this study it was mainly a random variation so that we could not calculate a conversion factor to standardize the filter paper results. A problem with collection of blood on filter paper is that the amount of reconstituted serum is variable. It depends on the subject's hematocrite and on several technical aspects of reconstitution[8] There are approaches to get around this variability. For example, in neonatal screening for PKU and hypothyroidism the test characteristics are set to a high sensitivity with the possibility of confirmation by a blood test of all positive cases[23] Such a two stage screening is probably not suitable for the diagnosis of acute infectious diseases such as dengue.

**Figure 1:**
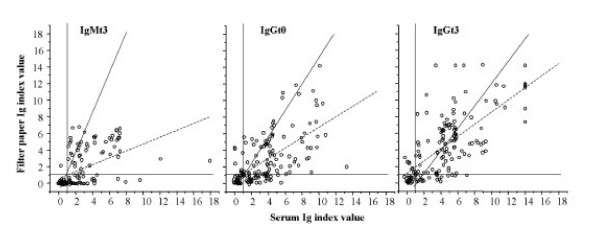
**Comparison of dengue ELISA in serum and reconstituted filter paper blood**. Serum and blood spots on filter papers were collected simultaneously from the same patient and tested at the same time with the same ELISA. The reference lines indicate the cut off value for the index value (< 1: negative; ≥ 1: positive). The sloped lines indicate the regression of y to x (^_____^) and x to y (-----).

Public health specialists are especially interested in early detection of outbreaks, notably of complicated dengue, which is mainly secondary dengue. This study shows that especially the value of dengue virus IgM detection on t0 and t3 with filter papers may lead to diagnostic misclassification and is thus of limited value for clinical confirmation. But, the results of measuring IgG in serum and filter papers eluates showed a rather good agreement with almost equal misclassification in both directions (Fig 1, panel C) Thus, with sufficient sample sizes and while taking cross reactivity with other flavivirusses, notably JEB virus, into account, dengue virus sero-prevalence studies, based on the detection of IgG antibodies eluted from filter papers, are a useful tool for epidemiologists.

## Conclusion

ELISA for detection of dengue antibodies in serum of febrile patients was not very precise and was subject to inter-laboratory variability. In this study the use of whole blood collected on filter papers introduced an extra, mainly random, error, affecting the diagnostic accuracy for the diagnosis of patients suspected of dengue virus infection. The correlation between serum IgG and IgG collected from whole dried blood was better and this method can be used for dengue virus IgG sero-prevalence studies.

## Competing interests

There is no conflict of interest. One of the co-authors, J. Groen, recently changed position, and now works for the company which produces the test kit which was used in this study. There has never been any contact between this company and the first author or the corresponding author. J. Groen, withdrew from this research project after his change of position and the results of this study and the contents of this paper were not available at the time that he changed position.

## Authors' contributions

T.T. Nga: Fieldwork and data collection, laboratory tests, datamanagement, analysis and statistics, writing manuscript

Peter J. de Vries: Study design, fieldwork and data collection, datamanagement, analysis and statistics, writing manuscript.

Hoang Lan Phuong: Fieldwork and data collection, datamanagement

Phan T. Giao: Fieldwork and data collection, datamanagement

L.Q. Hung: Fieldwork and data collection, datamanagement

Tran Q. Binh: Fieldwork and data collection, datamanagement, logistics and local scientific supervision, review and corrections of manuscript.

V.T.C. Mai: Supervision of laboratory work, review and corrections of manuscript

Nguyen V. Nam: Fieldwork and data collection, review and corrections of manuscript

Nico Nagelkerke: Study design, data analysis and statistics, writing manuscript

Piet A. Kager: Study design, review and corrections of manuscript

Jan Groen: Study design, laboratory tests, writing manuscript

## Pre-publication history

The pre-publication history for this paper can be accessed here:



## References

[B1] Guzman MG, Kouri G (2004). Dengue diagnosis, advances and challenges. Int J Infect Dis.

[B2] Vaughn DW, Nisalak A, Solomon T, Kalayanarooj S, Nguyen MD, Kneen R, Cuzzubbo A, Devine PL (1999). Rapid serologic diagnosis of dengue virus infection using a commercial capture ELISA that distinguishes primary and secondary infections. Am J Trop Med Hyg.

[B3] Groen J, Koraka P, Velzing J, Copra C, Osterhaus AD (2000). Evaluation of six immunoassays for detection of dengue virus-specific immunoglobulin M and G antibodies. Clin Diagn Lab Immunol.

[B4] Koraka P, Zeller H, Niedrig M, Osterhaus AD, Groen J (2002). Reactivity of serum samples from patients with a flavivirus infection measured by immunofluorescence assay and ELISA. Microbes Infect.

[B5] Innis BL, Nisalak A, Nimmannitya S, Kusalerdchariya S, Chongswasdi V, Suntayakorn S, Puttisri P, Hoke CH (1989). An enzyme-linked immunosorbent assay to characterize dengue infections where dengue and Japanese encephalitis co-circulate. Am J Trop Med Hyg.

[B6] Organization WH, Organization WH (1997). Laboratory Diagnosis. Dengue Haemorrhagic Fever Diagnosis, Treatment and Control.

[B7] Carlson MD (2004). Recent advances in newborn screening for neurometabolic disorders. Curr Opin Neurol.

[B8] Mei JV, Alexander JR, Adam BW, Hannon WH (2001). Use of filter paper for the collection and analysis of human whole blood specimens. J Nutr.

[B9] Vazquez S, Fernandez R, Llorente C (1991). Usefulness of blood specimens on paper strips for serologic studies with inhibition ELISA. Rev Inst Med Trop Sao Paulo.

[B10] Ruangturakit S, Rojanasuphot S, Srijuggravanvong A, Duangchanda S, Nuangplee S, Igarashi A (1994). Storage stability of dengue IgM and IgG antibodies in whole blood and serum dried on filter paper strips detected by ELISA. Southeast Asian J Trop Med Public Health.

[B11] Vazquez S, Saenz E, Huelva G, Gonzalez A, Kouri G, Guzman M (1998). Detection of IgM against the dengue++ virus in whole blood absorbed on filter paper. Rev Panam Salud Publica.

[B12] Hogrefe WR, Ernst C, Su X (2002). Efficiency of reconstitution of immunoglobulin g from blood specimens dried on filter paper and utility in herpes simplex virus type-specific serology screening. Clin Diagn Lab Immunol.

[B13] El Mubarak HS, Yuksel S, Mustafa OM, Ibrahim SA, Osterhaus AD, de Swart RL (2004). Surveillance of measles in the Sudan using filter paper blood samples. J Med Virol.

[B14] de Swart RL, El Mubarak HS, Vos HW, Mustafa OM, Abdallah A, Groen J, Mukhtar MM, Zijlstra EE, El Hassan AM, Wild TF, Ibrahim SA, Osterhaus AD (2001). Prevention of measles in Sudan: a prospective study on vaccination, diagnosis and epidemiology. Vaccine.

[B15] Cobelens FG, Groen J, Osterhaus AD, Leentvaar-Kuipers A, Wertheim-Van Dillen PM, Kager PA (2002). Incidence and risk factors of probable dengue virus infection among Dutch travellers to Asia. Trop Med Int Health.

[B16] Chanama S, Anantapreecha S, nuegoonpipat A, Sa-gnasang A, Kurane I, Sawanpanyalert P (2004). Analysis of specific IgM responses in secondary dengue virus infections: levels and positive rates in comparison with primary infections. J Clin Virol.

[B17] Vazquez S, Perez AB, Ruiz D, Rodriguez R, Pupo M, Calzada N, Gonzalez L, Gonzalez D, Castro O, Serrano T, Guzman MG (2005). Serological markers during dengue 3 primary and secondary infections. J Clin Virol.

[B18] Donoso MO, Lemmer K, Biel SS, Groen J, Schmitz H, Durand JP, Zeller H, Niedrig M (2004). Quality control assessment for the serological diagnosis of dengue virus infections. J Clin Virol.

[B19] Guzman MG, Pelegrino JL, Pumariega T, Vazquez S, Gonzalez L, Kouri G, Arias J (2003). Quality control of the serological diagnosis of dengue in laboratories throughout the Americas, 1996-2001. Rev Panam Salud Publica.

[B20] Kuno G, Cropp CB, Wong-Lee J, Gubler DJ (1998). Evaluation of an IgM immunoblot kit for dengue diagnosis. Am J Trop Med Hyg.

[B21] Wright PF (1998). International standards for test methods and reference sera for diagnostic tests for antibody detection. Rev Sci Tech.

[B22] Fenollar F, Raoult D (1999). Diagnosis of rickettsial diseases using samples dried on blotting paper. Clin Diagn Lab Immunol.

[B23] Van Vliet G, Czernichow P (2004). Screening for neonatal endocrinopathies: rationale, methods and results. Semin Neonatol.

